# Vascular segmentation in hepatic CT images using adaptive threshold fuzzy connectedness method

**DOI:** 10.1186/s12938-015-0055-z

**Published:** 2015-06-19

**Authors:** Xiaoxi Guo, Shaohui Huang, Xiaozhu Fu, Boliang Wang, Xiaoyang Huang

**Affiliations:** Computer Science Department, Xiamen University, Xiamen, China; Computer Engineering College, Jimei University, Xiamen, China

**Keywords:** Hepatic vessel segmentation, Fuzzy connectedness method, Fuzzy affinity, Adaptive threshold

## Abstract

**Background:**

Fuzzy connectedness method has shown its effectiveness for fuzzy object extraction in recent years. However, two problems may occur when applying it to hepatic vessel segmentation task. One is the excessive computational cost, and the other is the difficulty of choosing a proper threshold value for final segmentation.

**Methods:**

In this paper, an accelerated strategy based on a lookup table was presented first which can reduce the connectivity scene calculation time and achieve a speed-up factor of above 2. When the computing of the fuzzy connectedness relations is finished, a threshold is needed to generate the final result. Currently the threshold is preset by users. Since different thresholds may produce different outcomes, how to determine a proper threshold is crucial. According to our analysis of the hepatic vessel structure, a watershed-like method was used to find the optimal threshold. Meanwhile, by using Ostu algorithm to calculate the parameters for affinity relations and assigning the seed with the mean value, it is able to reduce the influence on the segmentation result caused by the location of the seed and enhance the robustness of fuzzy connectedness method.

**Results:**

Experiments based on four different datasets demonstrate the efficiency of the lookup table strategy. These experiments also show that an adaptive threshold found by watershed-like method can always generate correct segmentation results of hepatic vessels. Comparing to a refined region-growing algorithm that has been widely used for hepatic vessel segmentation, fuzzy connectedness method has advantages in detecting vascular edge and generating more than one vessel system through the weak connectivity of the vessel ends.

**Conclusions:**

An improved algorithm based on fuzzy connectedness method is proposed. This algorithm has improved the performance of fuzzy connectedness method in hepatic vessel segmentation.

## Background

In the diagnosis and study of vascular-related diseases, the structure and morphology of the hepatic vessels provide vital information. Especially for surgical planning and outcome assessment of liver operations, such as living-related liver transplants and oncologic resections, it is crucial to present a patient-individual 3D structure of the liver along with its vasculature and lesions. Therefore, to achieve an accurate and robust extraction of the intrahepatic vessels is an essential step and plays an important role in medical image analysis.

Due to the low contrast between the blood vessels and the surrounding liver parenchyma, the complex morphology as well as the pathologies of the hepatic vessel system, vascular segmentation is a challenging task and has gained increasing attention and interest. Several specific methods have been proposed to segment vessels in hepatic CT images in recent years [1–5]. Most methods are histogram based, region growing based, level-set based, and geometrical model based.

A theory of fuzzy objects for n-dimensional digital spaces based on a notion of fuzzy connectedness of image elements and algorithms for extracting a specified fuzzy object were presented in [6]. Since then, this fuzzy connectedness framework and its extensions have been extensively utilized in many medical applications. Based on an improved fuzzy connectedness algorithm, Harati et al. developed a fully automatic and accurate method for tumor region detection and segmentation in brain MR images [7]. Lloréns et al. used fuzzy-connectedness and morphological processing to segment jaw tissues in dental 3D CT images [8]. Ciesielski et al. jointed graph cut and relative fuzzy connectedness to segment bones from CT images [9]. Badura et al. combined fuzzy connectedness and the evolutionary computation to segment 3D lung nodule [10]. When using fuzzy connectedness method in liver vessel segmentation task, two problems occurred. One is the excessive computational cost, and the other is the difficulty of choosing a proper threshold value for final segmentation. The work in Ref. [11] focused on minimize the computation of fuzzy connectedness relations by pre-determined thresholds for fuzzy objects. However, this pre-determined threshold method is not applicable in hepatic vessel segmentation. In this paper, we present an accelerated strategy to reduce the connectivity scene calculation time and an adaptive threshold based on watershed-like method to classify the connectivity scene in “Methods”. The materials utilized and the experiment results are detailed in “Results”. We state our conclusion in “Conclusions”.

## Methods

### Fuzzy adjacency and affinity

We refer to a 3D hepatic CT image as a scene *C*. The voxels in CT image have a fuzzy adjacency relation, denoted by *α*. The adjacency relation assigns every pair (*c*, *d*) of voxels a value between zero and one, denoted by *μ*_*α*_(*c*, *d*). The closer *c* and *d* are spatially to each other, the greater is this value. In this paper, we used the 6-adjacency relation for *α*. That is,

1 $$ \mu_{\alpha } (c,d) = \left\{ \begin{array}{l} 1, \quad if\sqrt {\sum\limits_{i} {(c_{i} - d_{i} )^{2} } } \le 1 \hfill \\ 0, \quad {\text{ otherwise}}. \hfill \\ \end{array} \right. \, $$

Another local fuzzy relation called fuzzy affinity is denoted by *k*. For any voxels *c* and *d*, the strength of this relation, denoted by *μ*_*k*_(*c*, *d*), lies between zero and one, and indicates how the voxels “hanging together” locally in the scene. The general form of *μ*_*k*_(*c*, *d*) is

2 $$ \mu_{k} (c,d) = \, \mu_{\alpha } (c,d) \, [\omega_{1} h_{1} (f(c),f(d)) \, + \omega_{2} h_{2} (f(c),f(d))], \, $$

where *f*(*x*) is the Hounsfield unit (HU) scale of voxel *x*; *h*_1_ and *h*_2_ are scalar-valued functions with range[0, 1]; *ω*_1_ and *ω*_2_ are free parameters satisfying:

3 $$ \omega_{1} + \omega_{2} = 1 $$

Through tests and comparisons, we chose $h_{1} (f(c),f(d)) = \, e^{{ - \frac{1}{2}\left[ {{{\left( {\frac{f(c) + f(d)}{2} - m} \right)} \mathord{\left/ {\vphantom {{\left( {\frac{f(c) + f(d)}{2} - m} \right)} s}} \right. \kern-0pt} s}} \right]^{2} }}$, *ω*_1_ = 1, and *ω*_2_ = 0 in our study. Hence, the affinity relation in this paper is

4 $$ \mu_{k} (c,d) = \, \mu_{\alpha } (c,d) \, \cdot e^{{ - \frac{1}{2}\left[ {{{\left( {\frac{f(c) + f(d)}{2} - m} \right)} \mathord{\left/ {\vphantom {{\left( {\frac{f(c) + f(d)}{2} - m} \right)} s}} \right. \kern-0pt} s}} \right]^{2} }} , $$

where *m* and *s* represent the mean and standard deviation of voxel values in the object of interest.

### Fuzzy connectedness

The global fuzzy relation on voxels is called fuzzy connectedness, denoted *K*. The strength of this relation *μ*_*K*_(*c*, *d*) lies between zero and one. To assign a connectedness value to any pair of voxels (*c*, *d*) needs taking all possible paths between *c* and *d* into consideration. Each path is a sequence of links formed between successive voxels, starting from *c* and ending in *d*. The strength of a path is the weakest pairwise voxel affinity in it. And the fuzzy connectedness strength between *c* and *d* is the strength of the strongest of all paths. Assuming that there are *n* paths (*p*_*cd*_^1^, *p*_*cd*_^2^, …, *p*_*cd*_^*n*^) from *c* to *d*. A path *p*_*cd*_^*i*^ consists of voxels < *c*, *a*^(1)^, *a*^(2)^, …, *a*^(*m*)^, *d* >.

The strength of a path *μ*_*K*_(*p*_*cd*_^*i*^) and connectedness of (*c*, *d*) are described as follows:

5 $$ \mu_{K} (p_{cd}^{i} ) = \hbox{min} \left[ {\mu_{k} (c,a^{(1)} ),\mu_{k} (a^{(1)} ,a^{(2)} ), \ldots ,\mu_{k} (a^{(m)} ,d)} \right] $$

6 $$ \mu_{K} (c,d) = \hbox{max} \left[ {\mu_{K} (p_{cd}^{1} ),\mu_{K} (p_{cd}^{2} ), \ldots ,\mu_{K} (p_{cd}^{n} )} \right]. $$

### The parameters for affinity relation

The fuzzy segmentation result is highly correlated to the mean value *m* and standard deviation value *s* in (4). Therefore, it is the most important step to obtain these parameters before starting a connectivity scene calculation. Most current algorithms used a relatively small space around the seed to estimate *m* and *s* for the intended object, hence the *m* and *s* are sensitive to the location of the seed. Due to the low contrast between the blood vessels and liver parenchyma, and the small vessel diameter, the manual selection of the seed may have great impact on whether the segmentation result is accurate. For example, the results from a seed in the center of the vessel and the one on the edge are distinguishing.

To reduce the influence of the seed position and enhance the robustness of fuzzy connectedness method, we use a cube (20 × 20 × 20 voxels) to enclose the seed in the center of it. In this cube, it would contain vessel as well as other tissues such as liver parenchyma. The Ostu algorithm [12] is utilized to achieve a threshold to classify all voxels in the cube into two groups (the vessels and others). Then *m* and *s* of the vessel group are calculated and we replace the value of the seed with *m*.

### A lookup table

During the connectivity scene calculation, the computational cost is determined mainly by evaluation of affinity relations. As the HU values of vessels are discrete and always lie in 100–230, same voxel pairs would be calculated repeatedly for many times. To speed up this procedure, a lookup table is introduced. At the beginning of fuzzy connectedness algorithm, a table of 256 × 256 would be filled at first. This table contains the *μ*_*k*_(*c*, *d*) of the voxel pair (*c*, *d*) with HU value (*f*(*c*), *f*(*d*)) ranging from (0, 0) to(255, 255), which covers all the possible combination of vessel voxel pairs. Every time when the value of *μ*_*k*_(*c*, *d*) is needed, it can just be retrieved from the lookup table and does not need to be calculated again.

### The algorithm based on fuzzy connectedness method for hepatic vessel segmentation

The fuzzy connectedness algorithm for vascular segmentation needs the specification of a seed to build the affinity relations between other voxels and itself, and then to calculate a connectivity scene. Using a threshold, it can produce a segmentation result.

The algorithm based on fuzzy connectedness method for hepatic vessel segmentation is presented as follows:

*Input: a scene C, a seed S*

*Output: vessel segmentation result V*

*Auxiliary Data Structures: A 3D array representing the connectivity scene CS and a queue Q contains voxels to be processed.*

*Begin*

*1 establish the lookup table;*

*2 set all voxels of CS to 0 except S which is set to 1;*

*3 push S to Q;*

*4 while Q is not empty do*

*5 remove a voxel c from Q for which CS(c) is maximal;*

6 for each voxel e such that *μ*_*k*_(*c*, *e*) > 0 do

7 set f_min = min{CS(c), *μ*_*k*_(*c*, *e*)};

*8 if f_min* > *CS(e) then*

*9 set CS (e)* = *f_min;*

*10 if e is already in Q then*

*11 update the location of e in Q;*

*12 else*

*13 push e in Q;*

*14 end for*

*15 end while*

*16 determine a threshold T*

*17 set V* = *segment CS by T;*

*End*

### Adaptive threshold

In the step 16 of the algorithm, a threshold *T* is used to filter the connectivity scene *CS* and generate the final result. Currently this threshold can only be preset by users. To choose the threshold automatically, the histogram of *CS*, *H*(*x*), should be calculated first. Given a threshold *h*, total volume of segmented object of interest can be expressed as follows:

7 $$ volume(h) = voxelsize*\int_{h}^{1.0} {H(x)dx} $$

The CT values of hepatic vessel were very similar and this makes them “hanging together” easily and results in obvious peaks in the histogram. The proper threshold can be calculated from these peaks. The idea of adaptive threshold searching method is as follows: the affinity value of scene represents the similarity of voxels; a high affinity value means a voxel is more similar to the seed. Because the seed lies in the vessel, voxels lying in the same vessel may achieve similar high affinity value. This feature would present as an obvious peak in the histogram. The terminals of blood vessels would reflect a weak connectivity of different vessels in liver CT image, so the histogram may contain many valleys before next peak appears. Another peak indicates that the connection has spread into another vessel. This procedure would be stopped when it reaches the possible maximum volume of hepatic vessels.

Based on the manual measurements of hepatic portal vein from 10 CT datasets shown in Table 1, the average volume of hepatic portal vein is 11.578 ± 1.684 ml.

**Table 1:** Statistics of Hepatic portal vein volume

Dataset	Voxel size (mm)	Num of hepatic portal vein voxels	Volume of hepatic portal vein (ml)
1	0.671 × 0.671 × 1	24,904	11.213
2	0.702 × 0.702 × 1	23,297	11.481
3	0.677 × 0.677 × 1	22,370	10.253
4	0.637 × 0.637 × 1	30,421	12.344
5	0.61 × 0.61 × 1	31,539	11.736
6	0.782 × 0.782 × 1	16,179	9.894
7	0.663 × 0.663 × 1	24,073	10.582
8	0.702 × 0.702 × 1	27,623	13.613
9	0.782 × 0.782 × 1	22,304	13.640
10	0.668 × 0.668 × 1	24,714	11.028

According to the volume of hepatic portal vein and taking hepatic vein, artery and portal vein into consideration, 50 ml is chosen as the maximum volume of hepatic vessels. To find the proper threshold, a watershed-like method is used in our study. Based on above description, the adaptive threshold finding method is presented as below.

*Input: a connectivity scene CS*

*Output: a suggested threshold T*

*Auxiliary Data Structures: An array H representing the histogram of CS*

*Begin*

*1 calculated H from CS;*

2 found an endpoint EP from H, where EP = max(h) and satisfies*volume*(*h*) ≥ 50 *ml*

*3 if EP is a peak then*

*4 let T* = *EP*

*5 else*

*6 search from EP to 1.0 until find a peak P;*

*7 let T* = *P;*

*8 endif*

*End*

## Results

Our algorithm was implemented in C++, and tests were performed with 4 different sizes of datasets acquired by different CT machines. Testing computer runs Windows XP, which has an Intel Core i5 CPU and 4 GB of main memory.

Table 2 lists the image datasets information used in our experiments and the performance of the implementation. It shows that the lookup table can speed up the computation of fuzzy connectedness relations by a factor of above 2.

**Table 2:** Dataset information and segmentation performance using fuzzy connectedness method without/with a lookup table

Dataset	I	II	III	IV
Data size (voxel)	(512, 512, 155)	(512, 512, 165)	(512, 512, 191)	(512, 512, 221)
Voxel size (mm)	0.683 × 0.683 × 1	0.702 × 0.702 × 1	0.613 × 0.613 × 1	0.637 × 0.637 × 1
Running time without a lookup table(s)	158	383	209	278
Running time with a lookup table(s)	74	160	97	119
Speedup factor	2.14	2.39	2.15	2.34
Seed location	(165, 221, 99)	(197, 190, 108)	(158, 229, 95)	(189, 233, 137)
Suggested threshold	0.89	0.779	0.718	0.775

A refined region-growing algorithm (RGG) presented in [1] has been widely used for intrahepatic vessel segmentation. Starting with a seed of the vessel with intensity *θ*_*beg*_, RRG iteratively accumulates the 26 adjacent voxels with an intensity equal to or greater than *θ*_*beg*_ and keeps them in a list *L*(*θ*_*beg*_), and then uses *L*(*θ*_*beg*_) as new seeds to collect all adjacent voxels with intensity greater than or equal to *θ*_*beg*_ – 1 in a list *L*(*θ*_*beg*_ − 1) until a given intensity *θ*_*end*_ is reached that creates voxels *L*(*θ*_*end*_) outside the vessel. From *θ*_*end*_ to *θ*_*beg*_, the number of voxels *N*(*θ*) is decreasing and changes considerably at *θ*_*opt*_ for most voxels belong to liver tissue are collected for thresholds below *θ*_*opt*_. RRG uses the threshold *θ*_*opt*_ for vessel segmentation. RRG’s segmentation result is high related to the starting seed’s location, and its running time mostly depends on *θ*_*beg*_ and *θ*_*end*_.

**Figure 1:**
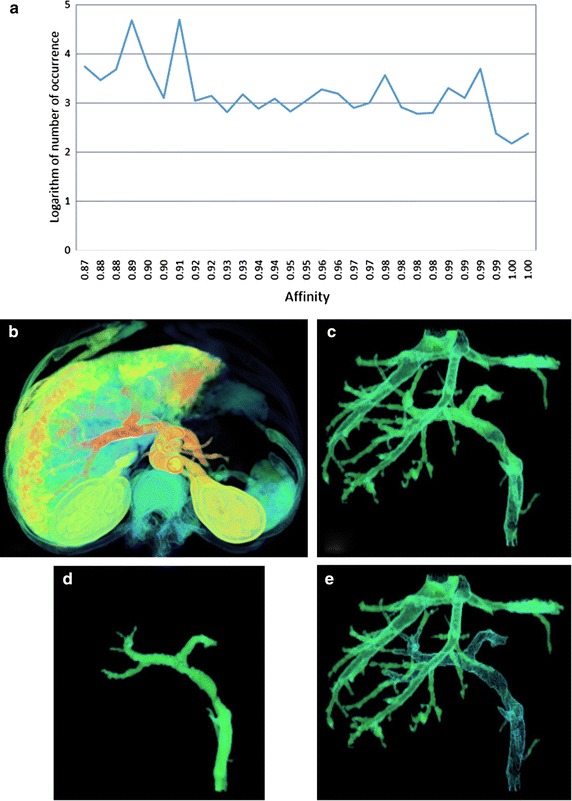
Segmentation results of dataset I: **a** histogram of the connectivity scene, **b** the fuzzy connectivity scene, **c** segmentation result using our method, **d** segmentation result using RRG, **e** the difference set *C.*

**Figure 2:**
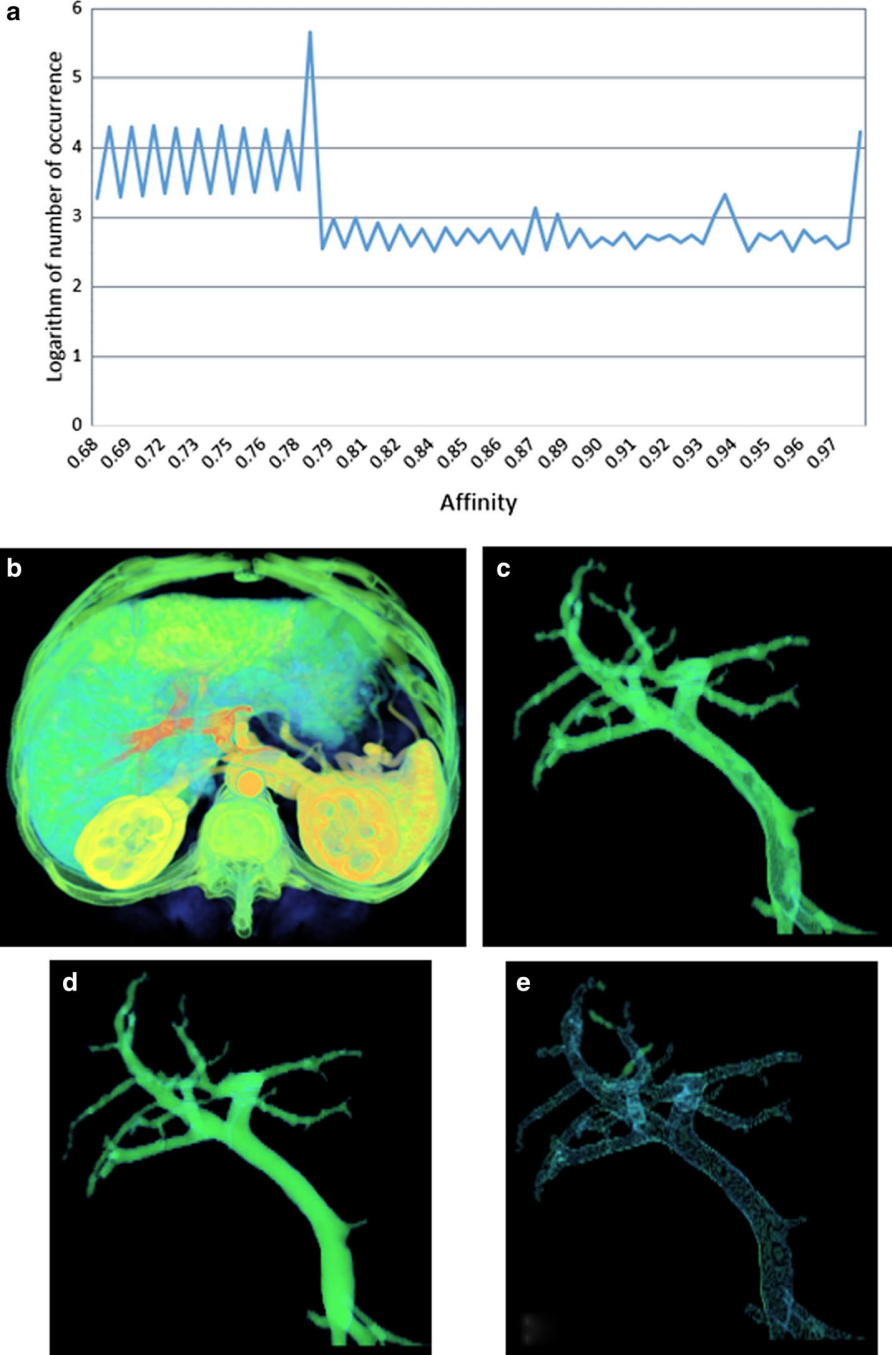
Segmentation results of dataset II: **a** histogram of the connectivity scene, **b** the fuzzy connectivity scene, **c** segmentation result using our method, **d** segmentation result using using RRG, **e** the difference set *C.*

**Figure 3:**
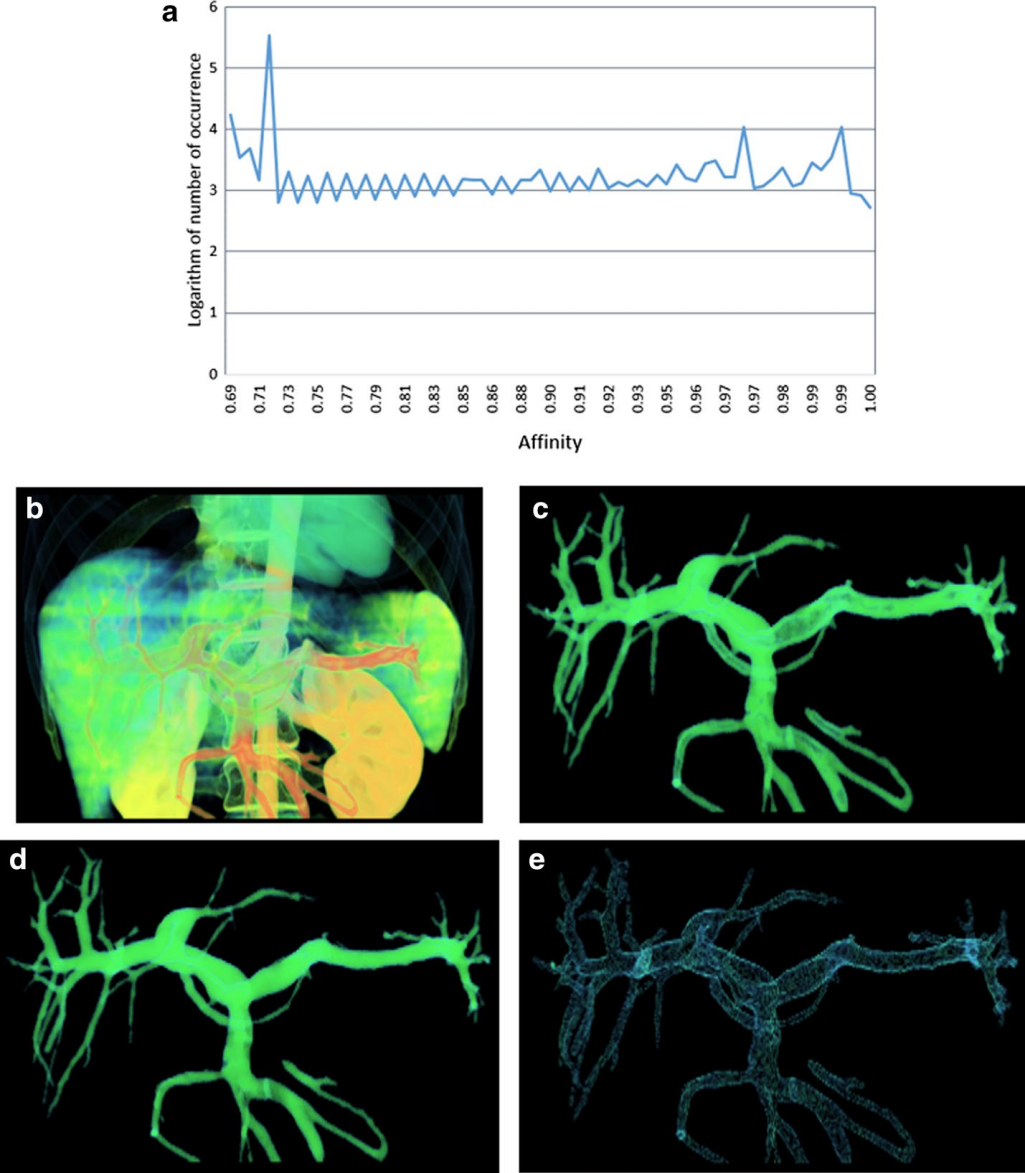
Segmentation results of dataset III: **a** histogram of the connectivity scene, **b** the fuzzy connectivity scene, **c** segmentation result using our method, **d** segmentation result using using RRG, **e** the difference set *C.*

**Figure 4:**
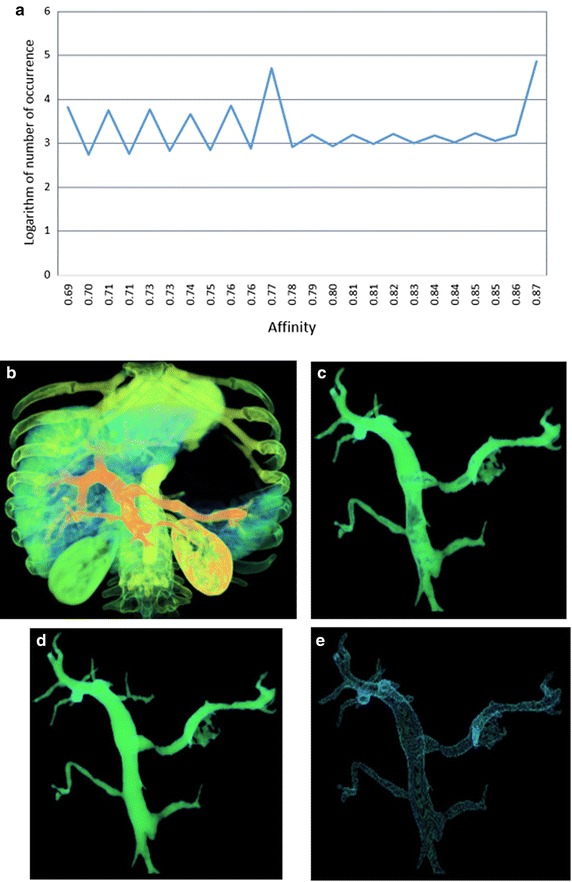
Segmentation results of dataset IV: **a** histogram of the connectivity scene, **b** the fuzzy connectivity scene, **c** segmentation result using our method, **d** segmentation result using using RRG, **e** the difference set *C.*

We applied RRG to these four datasets using the same seeds. Table 3 shows the quantitative comparisons between RRG and our method. Let *A* be the segmentation result using our method and *B* be the segmentation result using RRG, a different set *C*is the result of *A* − *B* as shown in Figures 1e, 2e, 3e and 4e. According to the set *C*, our method based on fuzzy connectedness has advantage of detecting vascular edge. Due to the influence of partial volume effect, the edge of vessel is blurred and mixed with other tissues such as liver parenchyma, and HU values of vessels decrease from center to the edge. Therefore, it is hard for RRG to collect the edge voxels that should be belonged to the vessel. Besides, different vessel systems may have a weak connectivity in the vessel ends, thus it is able to generate more than one vessel system using only one seed as shown in Figure 1c. According to Table 3 and set *C*, it is concluded that our method has better segmentation outcomes than RRG.

**Table 3:** Comparisons between our method and the refined region-growing algorithm (RRG) presented in Ref. [1]

Dataset	I	II	III	IV
Data size (voxel)	(512, 512, 155)	(512, 512, 165)	(512, 512, 191)	(512, 512, 221)
Seed location	(165, 221, 99)	(197, 190, 108)	(158, 229, 95)	(189, 233, 137)
Num of segmented vessel voxels using our method	83,520	45,624	111,399	89,631
Num of segmented vessel voxels using RRG	28,918	43,781	99,117	83,467

Figures 1, 2, 3 and 4 represent the histograms of the connectivity scenes, the corresponding connectivity scenes, the vessel segmentation results using fuzzy connected method and RRG, and the difference set *C*. In the histogram figures, the X axis represents affinity values which range from 0 to 1, while the Y axis represents the occurrence number (for convenience, in the form of its logarithm instead) of corresponding affinity value.

## Conclusions

An improved algorithm based on fuzzy connectedness method was proposed in this paper. This algorithm uses an accelerated strategy based on a lookup table to reduce the cost of fuzzy connectedness calculation, and a watershed-like method for adaptive threshold searching automatically. Utilizing the replacement of the seed’s value with mean value *m*, the algorithm is able to reduce the sensitivity to the location of the manually selected seed and to gain more robustness. Experiments based on four different datasets demonstrate the efficiency of the lookup table method, which achieves a speed-up factor of above 2. And the results also show that the adaptive threshold obtained automatically can always generate correct segmentation results.

This algorithm has indicated an improvement in the performance of fuzzy connectedness method in hepatic vessel segmentation, but it is still not enough to achieve an interactive speed. A parallel version of the algorithm which uses Graphics Processing Unit (GPU) is currently being studied, and it is hopeful to achieve a faster speed, thus more efficiency.
